# A Review on the Design and Performance of Enzyme-Aided Catalysis of Carbon Dioxide in Membrane, Electrochemical Cell and Photocatalytic Reactors

**DOI:** 10.3390/membranes12010028

**Published:** 2021-12-27

**Authors:** Fatin Nasreen Ahmad Rizal Lim, Fauziah Marpani, Victoria Eliz Anak Dilol, Syazana Mohamad Pauzi, Nur Hidayati Othman, Nur Hashimah Alias, Nik Raikhan Nik Him, Jianquan Luo, Norazah Abd Rahman

**Affiliations:** 1 School of Chemical Engineering, College of Engineering, Universiti Teknologi MARA, Shah Alam 40450, Malaysia; fatinnasreenlim@gmail.com (F.N.A.R.L.); victorialiceliz@gmail.com (V.E.A.D.); syazana7932@uitm.edu.my (S.M.P.); nurhidayati0955@uitm.edu.my (N.H.O.); nurhashimah@uitm.edu.my (N.H.A.); raikhan7952@uitm.edu.my (N.R.N.H.); noraz695@uitm.edu.my (N.A.R.); 2 Catalysis for Sustainable Water and Energy Nexus Research Group, School of Chemical Engineering, College of Engineering, Universiti Teknologi MARA, Shah Alam 40450, Malaysia; 3 State Key Laboratory of Biochemical Engineering, Institute of Process Engineering, Chinese Academy of Sciences, Beijing 100190, China; jluo@ipe.ac.cn

**Keywords:** enzyme membrane reactor, enzyme immobilization, carbon dioxide reduction, photocatalytic, electrochemical

## Abstract

Multi-enzyme cascade catalysis involved three types of dehydrogenase enzymes, namely, formate dehydrogenase (FDH), formaldehyde dehydrogenase (FaldDH), alcohol dehydrogenase (ADH), and an equimolar electron donor, nicotinamide adenine dinucleotide (NADH), assisting the reaction is an interesting pathway to reduce thermodynamically stable molecules of CO_2_ from the atmosphere. The biocatalytic sequence is interesting because it operates under mild reaction conditions (low temperature and pressure) and all the enzymes are highly selective, which allows the reaction to produce three basic chemicals (formic acid, formaldehyde, and methanol) in just one pot. There are various challenges, however, in applying the enzymatic conversion of CO_2_, namely, to obtain high productivity, increase reusability of the enzymes and cofactors, and to design a simple, facile, and efficient reactor setup that will sustain the multi-enzymatic cascade catalysis. This review reports on enzyme-aided reactor systems that support the reduction of CO_2_ to methanol. Such systems include enzyme membrane reactors, electrochemical cells, and photocatalytic reactor systems. Existing reactor setups are described, product yields and biocatalytic productivities are evaluated, and effective enzyme immobilization methods are discussed.

## 1. Introduction

The concentration of carbon dioxide (CO_2_) in the atmosphere has been increasing since the industrial era began. The Global Carbon Project has reported that the amount of CO_2_ released into the atmosphere has increased by about 1% every year [1]. Recently, the highest concentration of atmospheric CO_2_ was recorded at 412.5 ppm [2]. It can be found that the majority of anthropogenic CO_2_ emissions come from the combustion of fossil fuels for energy production. Although renewable energy production is expanding rapidly nowadays, fossil fuels are still widely used as energy sources, and an abundance of greenhouse gases released into the atmosphere may lead to serious global warming. To confront the issue, the Paris Agreement was negotiated and signed in 2016. About 196 state parties participated in the agreement and agreed to make efforts to reduce greenhouse gas emissions so that net-zero emissions can be achieved by the second half of the 21st century and the rise in global temperature limited to a maximum of 1.5 °C [3].

Among all the methods for reducing CO_2_ emissions, the utilization of CO_2_ to produce fuels and chemicals is highly attractive because not only could it decrease CO_2_ emissions into the atmosphere, it could also reduce fossil fuel consumption for energy production. Since CO_2_ is an abundant, readily available, highly stable molecule, non-flammable and also non-toxic, it is favourable for use as a raw material in chemical processes [4,5]. There are several pathways available for the conversion of CO_2_ to valuable chemical products. Catalytic hydrogenation of CO_2_ to methanol (CH_3_OH) is one of the well-known CO_2_ fixation methods and the CH_3_OH produced could be further transformed into biodiesels through transesterification, though the process requires a metal catalyst to facilitate the reaction. Normally, Cu-based and Pd-based catalysts would be used to catalyze the hydrogenation of CO_2_. However, modification of the catalysts would be needed, as the pure catalysts have low stability and selectivity towards CH_3_OH. Additionally, to obtain a high yield of CH_3_OH at a large scale, high temperatures and pressures have to be applied [6]. Furthermore, while there is an electrochemical approach for catalyzing the reduction of CO_2_ to CH_3_OH, a high applied potential is needed to initiate the reaction, and the metal electrodes involved can be easily deactivated through oxidation [7,8]. Thus, it is worth shifting attention to a process that could provide a lower energy route, guarantee sustainability, and protect the environment.

**Figure 1:**
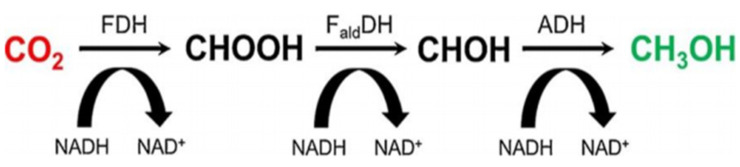
The biocatalytic pathway of the enzymatic conversion of CO_2_ to CH_3_OH reported by Obert and Dave [12].

The photocatalytic approach was introduced as an alternative method for catalyzing the reduction of CO_2_ to CH_3_OH, as it could provide a lower energy route by utilizing solar energy. Although the photocatalysts involved are abundant and easy to obtain [9], photocatalytic activity is still low for reducing CO_2_ [10,11]. Other than that, there is another attractive route that could be applied, namely, the biocatalytic pathway, which Obert and Dave [12] reported. This could replace the chemical catalytic route for converting CO_2_ into CH_3_OH—a process using three dehydrogenase enzymes: formate dehydrogenase (FDH), formaldehyde dehydrogenase (FaldDH), and alcohol dehydrogenase (ADH). The multi-enzymatic CO_2_ reduction also requires an equimolar electron donor, namely, nicotinamide adenine dinucleotide (NADH), to initiate the reaction. Three moles of NADH are needed to produce one mole of CH_3_OH in this multi-enzymatic cascade catalysis. The usage of enzymes is very appealing, as it offers a low temperature, low pressure route. Furthermore, due to the high selectivity of the dehydrogenases, the multi-enzymatic cascade catalysis could successfully be carried out in just one pot. It was noted that the FDH would first catalyze the transformation of CO_2_ to formic acid (CHOOH), then at the second stage the FaldDH enzyme would take part in the reduction of CHOOH to formaldehyde (CHOH), and finally the production of CH_3_OH would be catalyzed by ADH [13,14]. The detailed mechanism of cofactor (NADH)-aided CO_2_ reduction catalyzed by the dehydrogenase enzymes is still debated, but the number of studies focusing on the procedure for the first catalytic reaction step (CO_2_ + H^+^ + 2e^−^ ⇆ CHOOH) have grown in the past years. The redox potential (*E°′*) reported for the enzymatic transformation of CO_2_ to formate (HCOO^−^) and the oxidation of NADH to NAD^+^ are about −420 and −320 mV, respectively [15]. For the NADH-dependent FDH to start catalyzing a thermodynamically stable molecule of CO_2_, it needs to be first solubilized in water. Following Lewis acid-base theory, CO_2_ dissolves in water to form carbonic acid (H_2_CO_3_), reversibly. H_2_CO_3_ will be further dissociated into bicarbonate (HCO_3_^−^) and carbonate (CO_3_^2−^) ions [16]. It is suggested that FDH has a higher affinity towards hydrated derivatives of CO_2_ than gaseous forms. Commonly, the solubilization of CO_2_ in the reaction medium (buffer) is facilitated by carbonic anhydrase (CA). Then, the reduction of HCO_3_^−^ to CHOOH via FDH would be assisted by NADH oxidation. Finally, the FaldDH and ADH enzymes would consume the substrates produced (CHOOH and CHOH) and generate CH_3_OH at the end of the reaction. However, there are several challenges that would need to be overcome in order to help the system achieve high efficiency: the stability of the enzyme would need to be improved, enzyme and cofactor reusability would need to be enhanced, and a feasible, simple and efficient reactor setup that is suitable to operate the multi-enzymatic cascade system would have to be designed [17]. Figure 1 shows the biocatalytic pathway of the enzymatic conversion of CO_2_ to CH_3_OH reported by Obert and Dave [12].

The stability of the enzymes could be enhanced by employing enzyme immobilization. Obert and Dave [12] reported that encapsulating dehydrogenases in a porous silica sol–gel matrix could improve the biocatalytic productivity of the CO_2_ reduction. It was reported that the immobilized enzyme system was able to achieve a CH_3_OH yield of approximately 91.2%, while the CH_3_OH yield obtained by the free enzyme system was only approximately 43.8%. Now, various immobilization matrices and methods have been studied by researchers to achieve maximal reusability of biocatalysts and high yields of CH_3_OH production. In addition, due to the high cost of NADH, cofactor regeneration from its oxidized form, NAD^+^, is also essential for efficient, continuous CH_3_OH production through multi-enzymatic CO_2_ conversion. According to their findings, the biocatalytic reduction of CO_2_ via dehydrogenases and NADH as its cofactor has been widely applied in the enzyme membrane reactor (EMR) system. Recently, however, the implementation of enzyme immobilization and cofactor regeneration has been widely studied in electrochemical cell and photocatalytic reactor systems, and it has been proved that these systems are able to conduct the biocatalytic reduction of CO_2_ successfully. Nevertheless, the application of these biocatalytic reactors for CO_2_ conversion on a large scale is still limited, and there are many studies trying to improve the efficiency of the process. However, the EMR system has been widely applied for CO_2_ capture in industries and the incorporation of immobilized CA with membrane contactors has been gaining attention, as it is easy to scale up, enhances CO_2_ capture, and is economical, among other advantages. [18]. In past decades, CO_2_ conversion in electrochemical cells has only been applied on a laboratory scale and has not yet been successfully incorporated into industrial processes [19]. It is reported that the large overpotential and the low current density and stability have made it difficult to satisfy commercial demands [20]. The biocatalyzed artificial photosynthesis system is also still being studied, as the majority of the existing semiconductors or organic photocatalysts have low light absorption ability and reduction/oxidation potentials [21]. In this paper, the multi-enzymatic cascade conversion of CO_2_ in EMRs, electrochemical cells and photocatalytic reactor systems will be reviewed and the performance of each enzyme reactor system will be compared by observing the biocatalytic productivity attained by each system. The factors affecting the biocatalytic productivity of CO_2_ reduction in relation to obtaining high yields of CH_3_OH for future operations will be also evaluated.

## 2. Formate Dehydrogenase Catalyzing CO_2_ Reduction

Due to the advantages presented by the multi-enzymatic cascade system mentioned above, many researchers have worked to enhance the biocatalytic productivity of CO_2_ reduction. FDH has been recognized as a biocatalyst for the irreversible oxidation of formic acid (CHOOH) to CO_2_ [22]. Notably, there are several existing FDHs that have been proved capable of converting CO_2_ to CHOOH, reversibly. These can be categorized into two types, namely, metal-independent and metal-dependent FDHs (Table 1). It was found that the metal-independent/NAD^+^-dependent FDHs have lower catalytic turnover rates (k_cat_) for CO_2_ reduction to CHOOH than the metal-containing/NAD^+^-dependent FDHs. Several bacterial sources of metal-independent FDH enzymes have been found. FDHs from *Candida boidinii* (CbFDH) and *Thiobacillus* sp. (TsFDH) are the most widely applied metal-independent FDHs for catalyzing CHOOH production from CO_2_. The k_cat_ values reported for the two metal-independent FDHs are 0.015 and 0.32 s^−1^, respectively [23]. The low k_cat_ value provided by the FDH could be the reason for the low productivity obtained. There are also a few other metal-independent/NAD^+^-dependent FDH-producing microorganisms that have been reported, such as *Candida methylica*, *Myceliophthora thermophila*, and *Chaetomium thermophulum* [22]. The k_cat_ values of the FDHs for CO_2_ reduction are approximately 0.008, 0.1 and 0.023 s^−1^, respectively. Studies also showed that they have higher K_m_ value towards CO_2_ reduction than the metal-containing FDHs, indicating that they have lower CO_2_ affinity. Several types of intermediates were observed when CO_2_ was dissolved in aqueous solution, including the dissolved CO_2_, carbonic acid (H_2_CO_3_), carbonate (CO_3_^2−^), and bicarbonate (HCOO^−^). Among these intermediates, the FDH enzymes will be most attracted to the dissolved CO_2_ [24]. According to recent reports, one of the reasons for the low productivity of the biocatalytic reduction of CO_2_ is the slow hydration of CO_2_. Wang et al. [16] reported that the CHOOH production rate increased to 2.17 × 10^−3^ µmol/min when carbonic anhydrase (CA) was applied in the FDH-catalyzed CO_2_ reduction, while the initial CHOOH production rate without CA had achieved only about 4.17 × 10^−4^ µmol/min.

There are two kinds of metal containing-FDHs: the tungsten (W)- and molybdenum (Mo)-based FDHs. According to Tunney et al. [25], the metal-centered FDHs are widespread in anaerobic microorganisms. It has been reported that the metal-dependent FDH from *Syntrophobacter fumaroxidans* (SfFDH) has quite a high biocatalytic activity for reducing CO_2_, and the k_cat_ value reported for the CO_2_ reduction was in the range of 200–500 s^−1^, though the stability of the SfFDH could easily be affected by the presence of O_2_ [3,23]. On the other hand, it was noted that the Mo-containing/NAD^+^-dependent FDH from *Cupriavidus necator* (*C. necator*) is not sensitive to O_2_, and studies have also proved that it could catalyse the reduction of CO_2_ to CHOOH with a k_cat_ value of 10 s^−1^ [23]. In addition to this, Singh et al. [26] designed a microbial electrochemical system (MES) to generate CHOOH from CO_2_, in which they utilized *Escherichia coli* (*E. coli*), which is reported to have a metal-containing FDH. To maintain the growth of the biofilm, the *E. coli* was immobilized in iron phthalocyanine (FePc)-dispersed carbide-derived carbon (CDC) supported in an activated carbon fiber (ACF) electrode. The MES system yielded approximately 30 mg/L-h CHOOH and provided a Faradaic efficiency (FE) of approximately 58% at −1.0 V applied potential. Furthermore, Sakai et al. [27] proved that the W-containing/NAD^+^-dependent FDH from *Methylobacterium extorquens* AM1 produced a direct electron transfer (DET)-type bio-electrocatalytic wave at mesoporous carbon electrodes and catalyzed the interconversion of CO_2_/HCOO^−^ and NAD^+^/NADH successfully. The structure of the active sites in both Mo- and W-dependent FDHs are reported to be mostly similar [24]. There are two subunits (α and β) that can be found in the W-based FDHs. The α subunit contains a W-centered active site and at least one iron–sulfur (FeS) cluster, whilst the β subunit consists of a flavin mononucleotide (FMN) and an FeS cluster-binding motif. It was noted that the oxidation of NADH took place at FMN, and the electrons were further transferred to the W center via the FeS clusters then finally to CO_2_ in solution. Also of note, Yu et al. [23] developed a recombinant FDH for catalyzing the CO_2_ reduction to CHOOH by cloning the full length FDH from *C. necator* and expressing it in *E. coli* with a His-tag fused to the N-terminus of the γ subunit. When compared to the original isolated FDH from *C. necator*, the recombinant FDH has roughly 50% of its functionality. The recombinant FDH provided a k_cat_ value of 4.8 s^−1^ for CO_2_ reduction and 99 s^−1^ for CHOOH oxidation. Additionally, it could be further combined with glucose dehydrogenase (GDH) to support the continuous regeneration of NADH. Hence, CO_2_ reduction could be further improved by protein engineering. Not many studies have investigated the catalytic efficiency of the FaldDH and ADH enzymes towards CO_2_ reduction, though Luo et al. [17] reported that the reduction of CHOH to CH_3_OH catalyzed by ADH was found to be more efficient than the oxidation of CH_3_OH due to higher V_max_ values of 0.3 and 0.5 × 10^−3^ mM/min, respectively.

**Table 1:** FDH from different bacterial sources reported to be able to uptake CO_2_ directly.

FDH Sources	Classification	Efficiency towards CO_2_ Reduction	Ref.
FDH from *Candida boidinii* (CbFDH)	Metal-independent/NADH-dependent	The CbFDH was cloned and produced in *E. coli* BL21 (DE3). The k_cat_ values towards CO_2_ reduction reported for the soluble and immobilized recombinant CbFDH in polyvinyl alcohol (PVA) hydrogel were about 0.3183 and 0.0367 s^−1^, respectively.	[28]
FDH from *Myceliophthora thermophila* (MtFDH)	Metal-independent/NADH-dependent	The gene of MtFDH was produced, cloned, and expressed in *E. coli*. The k_cat_ obtained for the purified recombinant MtFDH when NaHCO_3_ was used as substrate was approximately 0.1 s^−1^.	[29]
FDH from *Chaetomium thermophilum* (CtFDH)	Metal-independent/NADH-dependent	CtFDH variants were expressed by transforming the plasmid libraries (G93-I94, R259, N120, and H312) into *E. coli* BL21 (DE3) cells. The k_cat_ values calculated were approximately 0.0317, 0.0867, 0.055, and 0.105 s^−1^ for CtFDH wild type, variant A1, variant A2, and variant B, respectively.	[30]
FDH from *Desulfovibrio desulfuricans* (DdFDH)	Mo-containing/NADH-independent	FDH was purified from *Desulfovibrio desulfuricans* under aerobic conditions [31]. The purified DdFDH was immobilized in a cellulose membrane on the surface of a pyrolytic graphite electrode. Using direct electrochemical method in the absence of mediators, the maximum current observed (via cyclic voltammetry) was around the potential of −250 mV during the first cycle for all the three methods applied to add CO_2_ into the solution, indicating that DdFDH could provide high electrocatalytic activity towards CO_2_ reduction.	[32]
FDH from *Escherichia coli* (EcFDH)	Mo-containing/NADH-independent	*E. coli* was immobilized on an iron phthalocyanine (FePc)-dispersed carbide-derive carbon (CDC) anode. The FePc–CDC-based microbial electrolysis system showed maximum HCOOH production and Faradaic efficiency (FE) of approximately 30 mg/L.h and 58%, respectively, at an applied potential of −1.0 V (Ag/AgCl) and continuous flow of CO_2_ at 120 mg/L.h.	[26]
FDH from *Cupriavidus necator* (CnFDH/FdsABG)	Mo-containing/NADH-dependent	To express the FdsABG FDH, the pTrc12HLB-FdsGBACD vector was transformed into *E. coli* DH5α cells. The FdsABG provided a k_cat_ value of 4.8 s^−1^ for CO_2_ reduction.	[23]
FDH from *Rhodobacter aestuarii* (RaFDH)	Mo-containing/NADH-dependent	RaFDH was heterologously expressed in *E. coli*. The recombinant RaFDH provided a k_cat_ value of approximately 0.805 s^−1^.	[33]
FDH from *Methylobacterium extorquens* AM1 (FoDH1)	W-containing/NADH-dependent	FoDH1 was absorbed on Ketjen Black (KB) modified with a glassy carbon electrode (GCE). The maximum current density recorded was approximately –0.30 mA cm^−2^.	[27]
FDH from *Syntrophobacter fumaroxidans* (SfFDH)	W-containing/NADH-independent	The isolated SfFDH was absorbed on the pyrolytic graphite electrode surface. The maximum current density recorded was approximately 0.08 mA cm^−2^ at pH 5.9, initial CO_2_ of 10 mM and applied potential of −0.8 V. The k_cat_ value calculated for CO_2_ reduction was 112 s^−1^.	[22,34]

## 3. Types of Enzymatic Reactor Systems Available for Biocatalytic Conversion of CO_2_

### 3.1. Enzyme Membrane Reactor (EMR) System

**Figure 2:**
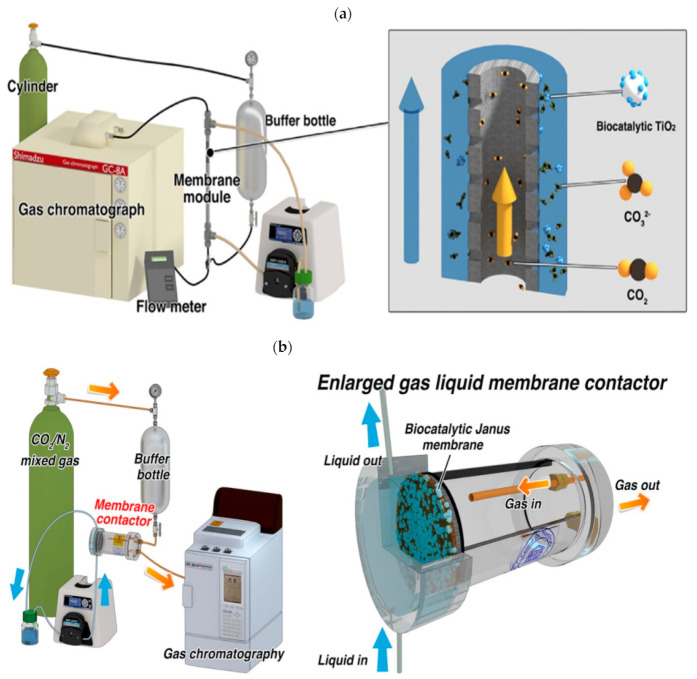
Schematic diagram of (**a**) the hollow fiber [36] and (**b**) flat-sheet Janus gas–liquid membranes contactors (GLMCs) [39] incorporated with encapsulated CA in TiO_2_ nanoparticles applied to catalyze CO_2_ hydration.

The concept of the EMR system is interesting; it is a combination of a membrane separation process together with enzymatic reactions. There are several EMR setup designs that could be applied for CO_2_ reduction. The multi-enzymatic cascade catalysis of CO_2_ involves two phases; the CO_2_ is in gas phase, whilst the enzymes are in liquid phase. Compared with conventional gas–liquid reactors, such as bubble columns and packed towers, EMR could provide great advantages for the enzymatic conversion of CO_2_, e.g., a large gas–liquid contact area, independent control of gas and liquid streams, and the fact that it is easy to operate and scale-up. Furthermore, the membrane acts as the non-selective interfacial barrier between the liquid and gas phases and ensures an efficient mass transfer process [35]. One of the EMR setups that has been studied by researchers for CO_2_ capture and hydration is the combination of immobilized carbonic anhydrase (CA) with a gas–liquid membrane contactor (GLMC). There are various types of membrane modules that could be utilized for the GLMC, such as hollow fiber, flat-sheet, tubular and spiral-wound membrane modules. Figure 2a,b show an example of a GLMC setup using hollow fiber membrane and flat-sheet membrane modules, respectively. The GLMC process is initiated by the gas absorption step, where the gas diffuses from the bulk gas phase to the gas–membrane boundary. After the gas reaches the gas–membrane boundary area, the gas permeates through the membrane pores and then transfers from the gas–liquid interface to the bulk liquid via physical or chemical absorption. Water (H_2_O) would normally be chosen as the absorbent for the GLMC process because it is environmentally friendly, economical, and highly compatible with polymeric membranes [36,37]. However, it has low CO_2_ hydration kinetics compared to chemical absorbents, such as amines, alkaline solvents, etc. Hence, to promote the CO_2_ hydration efficiency of the system, a biocatalyst, carbonic anhydrase (CA), is introduced to aid CO_2_ solubilization in the reaction solution. CA is known as a metalloenzyme that contains a zinc ion (Zn^2+^) at its active site, which allows it to catalyze the hydration of CO_2_ to bicarbonate (HCO_3_^−^) [38]. The final product, CH_3_OH or CHOOH, would then be removed via filtration from the intermediates formed during the reaction, which include NAD^+^ produced from the oxidation of NADH and the coproduct formed when a co-substrate is introduced into the reaction for NADH regeneration.

The gas–liquid interface is reported to be the most strategic location to place the enzyme, where the maximum CO_2_ concentration gradient and high mass transfer efficiency are expected. Furthermore, the membrane could not only effectively maintain a perfect gas–liquid interface but could also act as an ideal immobilization support for the enzymes involved, allowing them to retain their stability throughout the reaction and facilitating their reusability due to the strong porous structure and high surface area of the membrane. One of the challenges of the GLMC process, however, is membrane pore wetting, which could affect the mass transfer performance. One of the strategies that could be taken to overcome the challenge involves conducting hydrophobic surface modifications or hydrophobic membrane utilizations. Hou et al. [36] developed a superhydrophobic hollow fiber polypropylene (PP) membrane incorporating immobilized CA for CO_2_ hydration. The surface of the PP membrane was modified with titanium oxide (TiO_2_) nanoparticles to prevent membrane pore wetting which could result in high mass transfer resistance, and CA was covalently immobilized onto TiO_2_ nanoparticles using glutaraldehyde as the crosslinker. Better operational stability was observed for the superhydrophobic membrane compared to the pristine membrane. However, there were still losses of CO_2_ hydration efficiency throughout the reaction time for both membranes, which was likely caused by the accumulation of hydrated CO_2_ within the liquid and partial pore wettings. Although the hydrophobic membranes were able to maintain a defined gas–liquid interface, it is reported that hydrophilic membranes could ensure better enzyme immobilization than hydrophobic membranes. Thus, inspired by the Janus lipid bilayer structure, Gao et al. [39] had developed a Janus gas–liquid membrane reactor, involving hydrophilic and hydrophobic membranes, for CO_2_ hydration and transformation. The hydrophilic layer was introduced on the flat-sheet PP membrane surface by depositing it with polydopamine (PDA). After that, co-precipitation of TiO_2_ nanoparticles containing CA was carried out and FDH enzymes were immobilized on the fabricated Janus membrane. The flat-sheet Janus membrane was then placed vertically within the membrane cell with the hydrophilic part facing the liquid side, where the NADH with Tris-buffer solution was located. The immobilized enzymes on the hydrophilic surface were located near the gas–liquid interface, which allowed CO_2_ to immediately react with CA and be converted into HCOOH when it enters the liquid compartment. As predicted, the Janus reactor provided higher CO_2_ hydration efficiency and a higher HCOOH conversion rate compared to the original and unmodified membrane-based gas–liquid contactors. Rasouli et al. [37] also utilized a PP flat-sheet membrane for CO_2_ capture, but instead of encapsulating CA in TiO_2_ nanoparticles, co-deposition of PDA and polyethyleneimine (PEI) on the membrane surface was performed to support CA immobilization via glutaraldehyde. The abundance of amine functionalities on the polymeric membrane could offer more binding sites for the enzymes and promote enzyme loading. Moreover, the biocatalytic membrane showed great results for CO_2_ absorption, and thus demonstrated its promise for industrial application.

**Figure 3:**
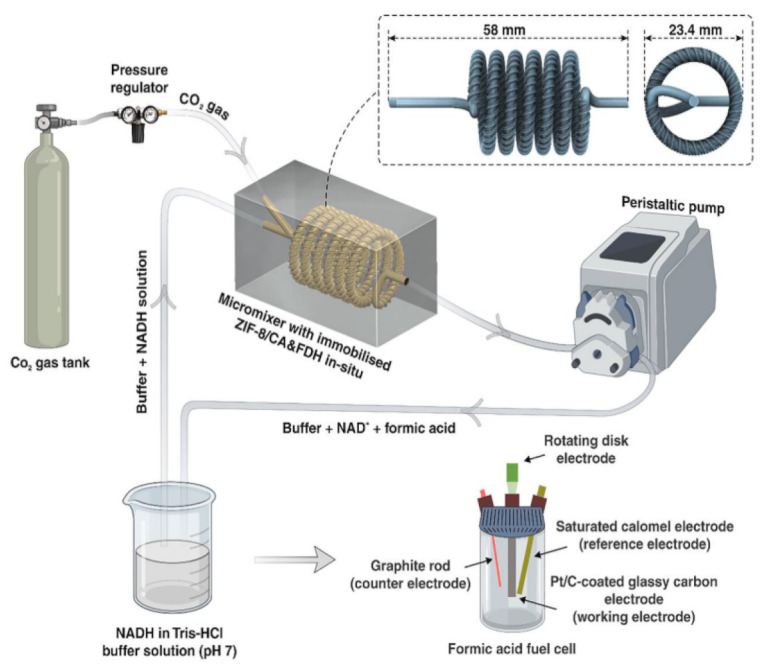
Schematic diagram of the micromixer setup used by Chai et al. [40] for the enzymatic cascade conversion of CO_2_ to HCOOH.

Moreover, recently, Chai et al. [40] introduced the idea of applying a micromixer with a threaded channel for CO_2_ conversion to HCOOH. The EMR setup utilized is as shown in Figure 3. It was reported that the utilization of the micromixer could enhance the mass transfer performance for the enzymatic cascade conversion of CO_2_ and allow better mixing of the dissolved gas in the liquid. The CA and FDH enzymes were biomineralized in ZIF-8 thin film, which was located on the surface of the PDA/PEI functionalized micromixer channel. Besides the unique properties of ZIF-8, such as a high specific area and high thermal and chemical stability, which makes it a good immobilization support for the enzymes, the imidazole group in ZIF-8 could also take part in the CO_2_ hydration process and yield bicarbonate. The CO_2_ gas and the cofactor enzyme (NADH) solution were fed into the micromixer through two separate channels. The hydration step of CO_2_ would take place first, where the active site of CA, which is the zinc-bound hydroxide, would attack the carbonyl bond of CO_2_ and form a metal-bound bicarbonate, which would then be displaced by a H_2_O molecule. The bicarbonate and NADH would bind at the FDH active site, which consists of the amino acid residues. With the bicarbonate as the substrate and NADH as the terminal electron donor, HCOOH would be produced from the cascade reaction. Besides ZIF-8, UiO-66 MOFs have also been tested for CO_2_ reduction in GLMC. The UiO-66-NH_2_ thin film was reported to be a robust immobilization support for CA and FDH enzymes, however, the HCOOH yield was found to be slightly higher by utilizing the ZIF-8 thin film, these being 3.7 μmol and 5.6 μmol, respectively [41]. Nevertheless, both UiO-66-NH2 and ZIF-8 biocatalytic membranes showed great potential for enzymatic applications involving high operating temperatures.

**Figure 4:**
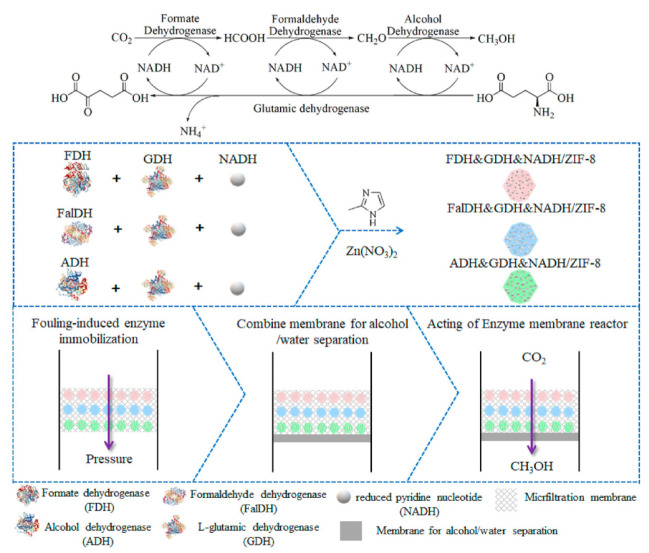
Ordered co-immobilization of a multi-enzyme cascade system with enzymes immobilized in metal organic framework in membrane [42].

Zhu et al. entrapped each of the three dehydrogenase enzymes with the cofactors and the cofactor-regenerating enzymes (glutamate dehydrogenase), together with ZIF-8, forming nanocomposites (Figure 4) [42]. These nanocomposites were then filtrated in a microporous PVDF membrane via a fouling-induced enzyme immobilization technique adopted from Luo and co-workers [43]. The setup enhanced the transformation of CO_2_ to methanol, and 50% of their original productivity was retained after 12 h in reaction.

**Figure 5:**
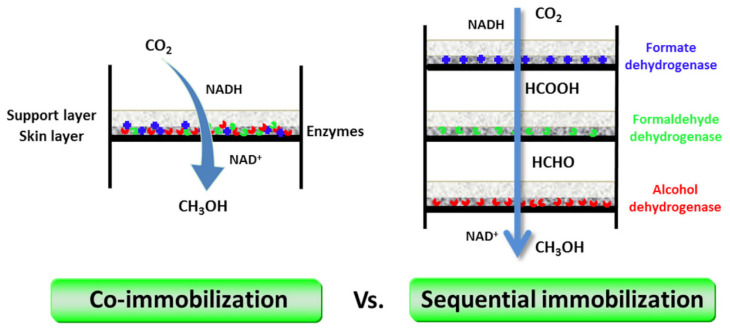
Enzyme membrane reactor setup for the production of methanol from CO_2_: co-immobilization versus sequential immobilization of enzymes in membranes [17].

Luo and co-workers introduced a membrane reactor setup which immobilized the three dehydrogenase enzymes, simultaneously or separately, in flat-sheet polymeric membranes (Figure 5) by simple pressure-driven filtration (i.e., by directing membrane fouling formation), without any addition of organic solvent [17]. Using this technique, the immobilization procedure is simple and facile [44,45,46], enzyme denaturation is minimized during immobilization, maximum enzyme loading is obtained without enzyme leakage during operation [47], and the product could be removed immediately from the enzyme active site in order to decrease product inhibition. From this research, it was found that co-immobilization did not improve methanol production compared with sequential immobilization because of the trade-off between the mitigation of product inhibition and low substrate concentration for the adjacent enzymes. The second enzyme (FaldDH) could not effectively consume the intermediate (formic acid) from the first reaction catalyzed by FDH. The reaction catalyzed by FaldDH is unfavorable because there is a chemical conflict between the required substrates (formic acid and NADH), and this reaction is sensitive to the substrate/product concentration and pH. Nevertheless, sequential immobilization could serve as an alternative to future multi-enzymatic cascade conversion of CO_2_ operations by allowing independent reaction conditions control for each enzymatic reaction stage and reducing the diffusion resistance between enzymes.

#### 3.1.1. Types of Membrane Used in Reactor Setups

Many researchers have studied the most robust membrane materials as the immobilization support for the enzymes to preserve their catalytic activity and stability. There are two types of membrane materials which are commonly applied in EMR: polymeric and ceramic membranes. Polymeric membranes are known to have good mechanical stability, biocompatibility, availability, and be relatively low in cost. The polymeric membranes can be grouped into two categories: hydrophilic and hydrophobic polymers. Hydrophilic polymers can incorporate attractions with water, including dipole–dipole interactions, hydrogen-bonding, and ion–dipole interactions, due to their abundance of functional groups (e.g., hydrogen bonds, amino groups, hydroxyl groups, carbonyl groups, etc.) [48]. Furthermore, the biocompatible functional groups on their surfaces also allow the easy, strong, and stable attachment of enzymes. There are a few examples of hydrophilic polymers available, namely, cellulose, polyamide, and polyimide membranes. Bacterial cellulose (BC) is one kind of nanocellulose that has been utilized for CO_2_ separation [49]. Dai et al. [50] reported several nanocellulose-based hybrid membranes and included a brief analysis of their performance respecting CO_2_ separation. It can be clearly seen that the modifications made to the cellulose membranes did help improve CO_2_ permeability and selectivity. It was noted that maximum CO_2_ selectivity could be obtained when the relative humidity of the feed gas is between 65% and 80%. However, although a further increase of relative humidity could potentially result in higher CO_2_ permeability, it could also mean lower CO_2_ selectivity. This is because the polymeric matrix tends to swell in the presence of water vapour, and this increases the spaces between nanocellulose fibers. It was suspected that gases other than CO_2_ might be able to pass through the cellulose membrane and thus cause the membrane to lose its selectivity. Hence, hybrid nanocellulose membranes could potentially show promising results for CO_2_ conversion in the future due to their high CO_2_ permeability and modification flexibility, allowing for an increase of CO_2_ adsorption sites, selectivity, and the chemical and physical properties of membranes).

The hydrophobic polymers which have been utilized for CO_2_ capture and conversion include polyvinylidene (PVDF), polyethylene (PE), polytetrafluoroethylene (PTFE), polypropylene (PP), and polysulfone (PSF) membranes. Interestingly, it was highlighted that blending hydrophilic and hydrophobic polymers could result in better chemical and thermal stability, the hydrophilic layer facilitating the enzyme immobilization process whilst the hydrophobic layer increases the mechanical strength of the membrane [37]. Recently, Guo et al. [51] studied the effect of cationic polyelectrolyte polyethyleneimine (PEI) on the performance of PE hollow fiber membranes (HFMs) and silica (SiO_2_) microspheres in FDH-catalyzed CO_2_ conversion to CHOOH. FDH was immobilized in both matrices. It was observed that the immobilized FDH in polydopamine (PDA)/PEI–SiO_2_ exhibited higher relative activity compared to immobilized FDH in PEI–polyacrylate (PAA)–PE—the values for which are 53.2% and 24.5%, respectively—after they have been reused for five cycles, proving that the PEI-modified SiO_2_ support is a more robust immobilization support than HFMs. Other than that, SiO_2_ is also known to have a higher CO_2_ adsorption ability than the PE HFM, and the abundance of amino groups provided by PEI and the PDA coating probably results in higher CO_2_ affinity for the composite than PEI–PAA–PE and the unmodified SiO_2_ microspheres.

A number of ceramic membranes have been applied in CO_2_ conversion, including titania, alumina, glass fiber, silicon, zeolites, zirconia. Their long service life and regeneration ability has motivated their application [52]. Inorganic supports are also reported to have higher mechanical strength and better resistance to operating conditions compared to organic supports [53]. However, the conversion of CO_2_ was still found to be low with polymeric and ceramic membranes.

Recently, many researchers have focused on applying mixed-matrix membranes (MMMs)—composites of organic and inorganic membranes—for CO_2_ separation and conversion. It has been noted that MMMs could provide higher permeability and selectivity than pure and unmodified polymeric or ceramic membranes. Soltani et al. [54] studied the effect of zinc oxide (ZnO) addition on the gas separation performance of a polyurethane (PU) membrane. It is reported that the PU membrane with 0.5 wt% ZnO content provided higher CO_2_ permeability than the pure PU membrane. The highest CO_2_ permeabilities for both membranes were observed when a 12 bar operating pressure was utilized, where the CO_2_ permeability of the PU–ZnO 0.5 wt% MMM and PU membrane were 80.72 Barrer and 69.09 Barrer, respectively. Moreover, Hosakun et al. [55] also modified the BC membrane with ZnO nanoparticles and studied its interactions with CO_2_, later comparing it with the results for the silk fibroin-modified and the basic BC membrane. The comparison showed that the basic BC membrane exhibited slightly higher CO_2_ permeability than the silk fibroin- and ZnO nanoparticles-modified BC membranes, the values for which were 2.73, 2.69, and 2.66 Barrer, respectively, at room temperature and with a feed pressure of 480 Pa. This is likely due to the presence of additional sites on the modified BC membranes. However, higher permeability could be expected from the BC-based membranes at higher feed pressures, since an already high permeability was obtained even when a low pressure was utilized. Thus, the BC-based MMMs could potentially be high-performing membranes for CO_2_ separation and conversion.

Metal–organic frameworks (MOFs) constitute another well-known variety of MMMs that have been developed. Zeolitic imidazolate frameworks (ZIFs) are the most studied MOFs due to their ultra-microporosity structure, high thermal and chemical stability, and high selectivity of CO_2_ [56]. Of late, several studies have incorporated ZIF-8–graphene oxide (GO) hybrid nanofillers with polymeric membranes and tested them for CO_2_ separation. Due to the high porosity and flexibility exhibited by the surface of ZIF-8 and polar functional groups provided by the GO, it can facilitate CO_2_ diffusion and thereby enhance CO_2_ permeability, solubility, and selectivity. Dong et al. [57] tested the CO_2_ separation performance of several MMMs (Pebax–ZIF-8@GO membranes) containing different amounts of ZIF-8@GO (0–8 wt%). It is reported that a Pebax–ZIF-8@GO membrane fabricated with 6 wt% ZIF-8@GO exhibited the best CO_2_ separation performance, where the CO_2_ permeability and CO_2_–N_2_ selectivity recorded were approximately 249 Barrer and 47.6, respectively. The high CO_2_ permeability provided by Pebax–ZIF-8@GO could be explained by the high porosity and flexibility possessed by ZIF-8, which would facilitate CO_2_ diffusion and lead to high CO_2_ permeability. The polar functional groups in GO could also allow specific interaction with CO_2_. Thus, the larger free volume introduced by ZIF-8@GO could further facilitate CO_2_ transport in the membrane. Pebax–ZIF-8@GO has the potential to be applied in biocatalytic CO_2_ reduction in the future, and several researchers have studied the application of ZIF-8 in enzyme immobilization. For example, Zhu et al. [42] embedded FDH, FaldDH, and ADH, together with NADH and a coenzyme (GDH), in ZIF-8, then co-immobilized the (enzyme and coenzyme)–ZIF-8 nanocomposites in the membrane. It is reported that the ordered (enzyme and coenzyme)–ZIF-8 in the membrane allow for a methanol yield approximately 2.8 times higher than (enzyme and coenzyme)–ZIF-8 in solution. The enzyme–ZIF-8 nanocomposites could also be added during the fabrication of the membrane. Hence, the mixed-matrix membrane involving ZIF-8 could be potentially used in a biocatalytic membrane reactor for multi-enzymatic CO_2_ conversion.

#### 3.1.2. Enzyme Immobilization Techniques in EMRs

Apart from the multiple choices of immobilization supports that could be used, there are also alternative methods for immobilizing the enzymes in EMRs, such as physical adsorption, covalent bonding, entrapment, encapsulation, cross-linking, etc. Numerous studies have conducted full reviews of the enzyme immobilization methods and updated their efficiency. Each of the methods has its own advantages and disadvantages. Physical adsorption is known as the simplest immobilization method of all, where the enzymes are normally attached to the membrane via Van der Waals forces, ionic bonds, hydrogen bonds, and hydrophobic bonds [18]. Although the activity of enzymes would be least affected by this method, it is most likely that enzyme leakage would occur [56]. The covalent bonding approach was later introduced as an alternative immobilization method, with which a stronger attachment of enzymes on the carrier could be obtained. However, several pre-treatment processes must be undergone before covalent coupling can be attained with the enzymes to promote functional groups, such as amine, hydroxyl, carboxyl, or epoxy groups, on the surface of the support, which would probably cause the method to be seen as tedious [48,56]. Crosslinking agents could be introduced to assist the covalent attachment of the enzymes on the membrane surface. For example, Rasouli et al. [37] utilized a covalent coupling method to immobilize carbonic anhydrase (CA) on a PP flat-sheet membrane with glutaraldehyde (GA) as a crosslinker, and used the biocatalytic membrane for CO_2_ capture. According to the results, the immobilized CA retained about 82.3% of its initial activity after 40 days of storage, whilst the free CA lost about 38% of its initial activity, thus proving that the covalent attachment method could preserve the relative activity of the enzymes well and further enhance the stability of CA. The biggest challenges with covalent bonding are that it affects the conformational shape of the enzymes and could also increase mass transfer resistances. For entrapment and encapsulation methods, the enzyme would be contained within the immobilization surface’s inner pores and the substrates would have to diffuse in to reach the enzymes while the products diffuse out of the pores after being produced, which could reduce product inhibition [58]. Moreover, it was suggested that the entrapment and encapsulation of enzymes be conducted in organic–inorganic hybrid microcapsules which could provide better protection for the enzymes from contaminants and extreme reaction conditions. In recent studies, many researchers have focused on ways to reduce mass transfer resistance and increase enzyme loading. It was reported that sequential (layer-by-layer assembly) co-immobilization of enzymes via entrapment and fouling-induced enzyme immobilization techniques could meet the objectives and show promising results for the biocatalytic conversion of CO_2_ with FDH, FaldDH, and ADH enzymes [42,59]. Hence, there is no doubt that the EMR system could have great advantages for the enzymatic conversion of CO_2_.

### 3.2. The Electrochemical Cell System

**Figure 6:**
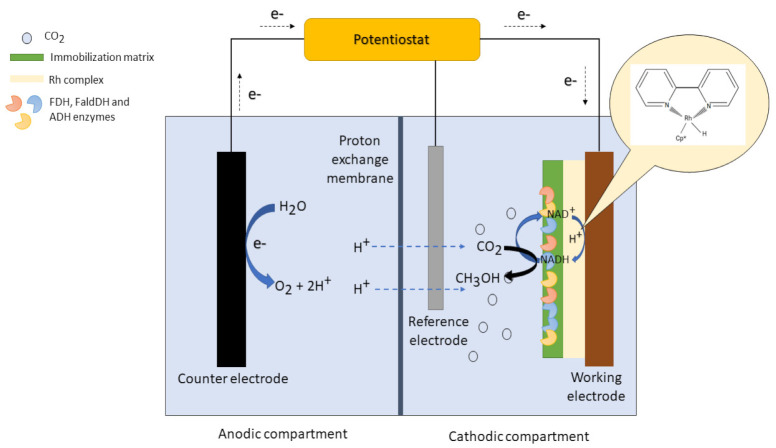
Schematic illustration of the general electrochemical cell design developed by researchers for catalyzing the enzymatic reduction of CO_2_, which consists of two types of compartments, namely, anodic and cathodic compartments. A Rh complex-modified working electrode was applied to prevent the production of undesired NAD_2_ dimers during the electrochemical regeneration of NADH and the dehydrogenase enzymes (FDH, FaldDH, and ADH) were immobilized on an immobilization matrix electrodeposited on the working electrode.

The electrochemical cell system is one of the reactor systems that could successfully catalyze the multi-enzymatic conversion of CO_2_. The ability to regenerate NADH using the electrochemical approach made the application of the enzymatic conversion of CO_2_ possible in the electrochemical cell system. There are several main components in the electrochemical cell system, including the electrolyte, the ion-exchange membrane, three types of electrodes (the working, counter, and reference electrodes), and two types of chambers (the anodic and cathodic chambers). Figure 6 shows the lab-scale electrochemical cell setup for conducting multi-enzymatic CO_2_ reduction. The working electrode would be immersed in the cathodic chamber, whilst the counter electrode would be soaked in the anodic chamber. The CO_2_ would first be saturated with H_2_O before being fed into the cathodic chamber. The catholyte having been saturated with CO_2_, the reduction of NAD^+^ would take place. The electrons generated from the oxidation of H_2_O at the anodic chamber would flow through the external circuit to reach the working electrode, while the hydrogen ions (H^+^) would be distributed from the anodic chamber to the cathodic chamber by the ion-exchange membrane to combine with NAD^+^ to form NADH [10]. The timing of the reduction of NAD^+^ would be controlled to obtain the desirable amount of NADH with which to initiate CO_2_ reduction. The solution would then be charged with the immobilized FDH, FaldDH, and ADH for CO_2_ conversion. At the end, CH_3_OH would be generated in the catholyte solution.

Besides being able to catalyze the direct electrochemical regeneration of NADH and the biocatalytic reduction of CO_2_ simultaneously, another unique feature of the electrochemical cell system is that it could also support enzyme immobilization and facilitate the reusability of the enzymes, like the EMR system. One of the techniques employed by the researchers to establish enzyme immobilization in the electrochemical cell system involves modifying the surface of the working electrode with the desirable immobilization support. For example, Schlager et al. [60] modified the carbon felt electrode with an alginate matrix and utilized it as the working electrode to immobilize dehydrogenase enzymes and catalyze the production of CH_3_OH. After 4 h of electrocatalysis at an applied potential of −1.2 V (vs. Ag/AgCl), the concentration of CH_3_OH obtained was around 0.15 ppm and the Faradaic efficiency (FE) achieved by the electrochemical system was approximately 10%. Chen et al. [61] encapsulated FDH enzymes in a metal–organic framework, NU-1006, and electrodeposited it on a Rh complex-modified fluorine-doped tin oxide (Rh-FTO) glass electrode to catalyze the CO_2_ reduction to CHOOH and electrochemical regeneration of NADH. It is reported that the Rh-FTO electrode could regenerate NADH effectively from NAD^+^ and the amount of CHOOH produced by the immobilized FDH system after 1 h at −1.1 V applied potential is higher than the free FDH system, the values for which are approximately 79 mM and 25 mM, respectively. Barin et al. [62] utilized modified electrospun polystyrene nanofibers (EPSNFs) in an immobilization matrix for retaining the activity of FDH. However, the immobilized FDH enzymes on the EPSNF matrix were not electrodeposited on the working electrode but instead were immersed in the reaction solution in the cathodic compartment after the reduction of NAD^+^ to NADH had been conducted at a certain hour. According to the report, the immobilized FDH retained about 53% of its initial activity after eight cycles. The biocatalytic productivity and FE recorded for the electrochemical system were 11.8 μM.mU^−1^·h^−1^ and 22.8%, respectively.

#### Challenges and Limitations of Biocatalytic CO_2_ Reduction in Electrochemical Cells

There are several challenges encountered when operating the biocatalytic reduction of CO_2_ in electrochemical cell systems, including the formation of dimers during the electrochemical reduction of NAD^+^ to NADH, high overpotential, high operational costs, low current density, and low chemical stability exhibited by the electrocatalysts [20,63]. There are several examples of metal electrodes that could be utilized to catalyze NADH regeneration, such as titanium (Ti)-, ruthenium (Rh)-, nickel (Ni)-, and platinum (Pt)-based electrodes [5]. The copper (Cu) foam electrode was later introduced as an alternative because it has a lower cost and is easy to fabricate [64]. Although utilizing Cu foam could successfully produce approximately 80% active NADH, the amount of inactive NADH produced is still considered high. This is because before the enzymatically active NADH can be regenerated from its oxidized formed (NAD^+^), NAD^+^ reduces to NAD radical (NAD∙) first. The NAD radical will then be further reduced and protonate to form 1,4-NADH. The unstable intermediate (NAD radical) is likely to lead to the formation of (NAD)_2_ dimers, which could further reduce to inactive NADH (1,6-NADH) instead of the active 1,4-NADH [65]. Normally, an electron mediator would be introduced to the electrochemical system to aid the electrochemical regeneration of NADH. The most used electron mediator is Rh (III). Recently, Song et al. [66] developed a strategy to obtain a high yield of 1,4-NADH without the application of an electron mediator which uses a carbon felt (CF) electrode on which Cu nanoparticles have been electrodeposited as the working electrode. The maximum NADH regeneration yield obtained by the system was about 92% when 2 mM CuSO_4_ was utilized, and it showed the highest efficiency for 1,4-NADH regeneration compared to the existing electrochemical NADH regeneration system.

Further studies are still needed to determine an economical electrode that could provide high electrical conductivity and stability for catalyzing NADH regeneration and CO_2_ reduction [19]. It has been reported that utilizing graphene oxide (GO) nanosheets as an electrocatalyst for CO_2_ reduction could increase the number of CO_2_ adsorption sites and further enhance electrocatalytic productivity. GO is a carbon-based material that has a large surface area (about 2630 m^2^/g), a mesoporous and microporous structure, good stability, and high conductivity [67,68]. It is also inexpensive compared to most metal catalysts. Furthermore, it could be easily modified with metal or non-metallic compounds to improve its adsorption properties [67]. Wu et al. [68] reported that the combination of bismuth (Bi) nanoparticles with GO nanosheets could give a Faradaic efficiency of approximately 92.1% at −0.97 V (vs. RHE) for the electrochemical reduction of CO_2_ to CHOOH. According to a TEM analysis, the Bi nanoparticles were uniformly disseminated on the GO nanosheets without any aggregation, indicating successful combination between the Bi nanoparticles and GO nanosheets. In addition to this, Dongare et al. [69] reported that doping graphene with nitrogen (N) could facilitate CO_2_ adsorption and that an N-doped graphene electrocatalyst provided a maximum Faradaic efficiency for CHOOH production of approximately 36.72% at −1.0 V (vs. RHE). Due to the unique characteristics possessed by GO, it is also able to act as an ideal enzyme immobilization support that could facilitate the reusability of enzymes for multiple operations [70]. Hence, graphene-based electrodes could be potent working electrodes to apply in the multi-enzymatic cascade reduction of CO_2_ in the future, as they could act as immobilization supports for the dehydrogenases involved and enhance CO_2_ adsorption.

### 3.3. Photocatalytic Reactor System

**Figure 7:**
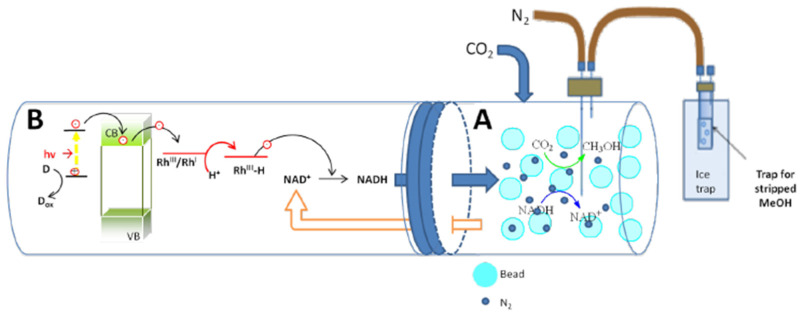
Biocatalytic reduction of CO_2_ to CH_3_OH via a photocatalytic reactor system [74].

The photocatalytic approach is one of the methods that could transform CO_2_ into value-added chemicals and fuels without requiring high inputs of energy and high operational costs. It is inspired by natural photosynthesis in plants, in which light energy is utilized to convert CO_2_ into carbohydrates and O_2_. Along with the electrochemical regeneration of NADH, the photochemical regeneration of NADH has been widely studied by researchers, as it could enable the development of a greener and more sustainable route than the electrochemical approach. To initiate the photochemical regeneration of NADH, similar to the electrochemical method, the process also requires a metal catalyst (semiconductor), an electron donor, and an electron mediator. There are various types of semiconductor materials that could be applied for NADH regeneration, such as titanium oxide (TiO_2_), cadmium sulfide (CdS), iron(III) oxide (Fe_2_O_3_), copper(I) oxide (Cu_2_O), indium vanadate (InVO_4_), etc. [71]. However, TiO_2_ is the most widely applied photocatalyst for catalyzing the regeneration of NADH due to its excellent catalytic properties, high stability, abundance, and low cost [11]. There are also several types of electron donors that can be utilized for the reaction, including EDTA, TEOA, and H_2_O. It has been reported that EDTA has the best interaction with TiO_2_ and better properties compared to TEOA and H_2_O [72]. Figure 7 shows the overall setup of the photocatalytic reactor system. To convert CO_2_ biocatalytically in a photocatalytic reactor system, first, the light harvesting step would take place, in which light would be absorbed by the semiconductor. For photocatalysis to occur, the amount of solar energy absorbed by the semiconductor must be higher than or equal to the band gap energy [73]. After the successful light absorption step, the electrons would be generated at the valence band (VB) and excite the conduction band (CB), leading to the production of the electron–hole pair. The photoexcited electrons would then move towards the outer surface of the semiconductor and be captured by the electron mediator. Next, the reduction of the mediator is expected to take place. The reduced form of mediator having reacted with NAD^+^, the oxidation of the mediator would occur and initiate the reduction of NAD^+^ to NADH. The oxidation of the electron donor would also take place due to the presence of the photoinduced holes. Finally, the photogenerated NADH would then be used to assist the multi-enzymatic conversion of CO_2_ to produce CH_3_OH.

#### Challenges and Limitations of Biocatalytic CO_2_ Reduction in Photocatalytic Reactors

Although the photocatalytic approach could offer a lower energy pathway, it is still difficult for the photocatalyst to achieve high productivity for CO_2_ reduction. This is because of the weak light utilization and redox ability possessed by the photocatalyst. One of the effective strategies developed to overcome the problem is by utilizing two-dimensional (2D)-layered materials as the photocatalyst. The large surface area hold by the 2D-layered material could provide more surface active sites and superior electron mobility for the photocatalytic reaction. Recently, Ji et al. successfully utilized a Z-scheme-based photocatalytic system to catalyze the biocatalytic reduction of CO_2_ to CHOOH [21]. With the utilization of hybrid black phosphorus (BP) and antimonene (AM) ultrathin nanosheets and the application of the Z-scheme electron transfer mechanism, the photocatalytic system is able to improve the separation efficiency of the electron–hole pairs, resulting in high redox potentials for the photochemical regeneration of NADH. According to the report, approximately 90% of NADH regeneration and 0.266 μmol CHOOH/mg U enzyme of biocatalytic productivity were achieved by the system. It has also been reported that the light absorption ability of the photocatalyst could be enhanced by modifying its surface with a photosensitizer. Aresta et al. modified TiO_2_ with a chromium(III) anionic complex, [CrF_5_(H_2_O)]^2−^, and utilized it as a photocatalyst to catalyze the photochemical regeneration of NADH. The [CrF_5_(H_2_O)]^2−^@TiO_2_ showed the highest 1,4-NADH regeneration yield compared to other photocatalysts tested for the reaction (Cu_2_O, InVO_4_, and rutin@TiO_2_) [74]. Enzyme immobilization was also introduced to the photocatalytic reactor system to retain the biocatalytic activity and stability of the enzymes for multiple operations. Gu et al. integrated the photocatalytic unit with a hollow fiber membrane (HFM) reactor. It was noted that after NADH was produced at the photocatalytic unit, it would then be fed to the immobilized FDH-HFM reactor to induce CHOOH production from CO_2_. The cycle continues as the reaction solution is fed back to the photocatalytic unit to reconvert NAD^+^ to NADH. Furthermore, the integrated photocatalytic system gives a higher CHOOH production compared to the unintegrated photocatalytic unit, the yield of CHOOH obtained with the systems being 1.04 mM h^−1^ and 0.14 mM h^−1^, respectively [72], proving that enzyme immobilization is a crucial step for achieving high productivity in the biocatalytic reduction of CO_2_.

## 4. Performance of the Enzymatic Reactor Systems towards the Multi-Enzymatic Conversion of CO_2_ to CH_3_OH

Table 2 shows a complete review of researchers working on the three enzymatic reactor systems for the multi-enzymatic reduction of CO_2_. The majority of EMR systems have employed enzyme immobilization and co-factor regeneration to enhance the performance of the multi-enzymatic cascade catalysis. There are many techniques of enzyme immobilization and kinds of support matrix that contribute to the efficiency of the EMR system. Among all the reactor systems listed in Table 2, several EMR systems have shown quite a high biocatalytic productivity for the enzymatic transformation of CO_2_. One of the EMR systems that achieved high biocatalytic productivity was a cationic polyelectrolyte-doped hollow nanofiber membrane reactor system utilized by Ji et al., which achieved a CHOOH yield of approximately 103.2%. In this system, Ji et al. [75] used glutamate dehydrogenase (GluDH) and glutamic acid as the enzyme and substrate, respectively, for in situ NADH regeneration and carbonic anhydrase (CA) to facilitating the hydration of CO_2_. The co-immobilization system containing oxidoreductases, GluDH, and CA achieved the highest CH_3_OH concentration compared to the other tested systems, such as the free enzyme system and the co-immobilization system without CA. Another high yield of CH_3_OH was obtained from the EMR system designed by Jiang et al. [76]—approximately 92.1%. In their study, Jiang et al. [76] proved that operating conditions could highly affect the biocatalytic productivity of the multi-enzymatic cascade CO_2_ conversion. The optimum reaction conditions reported for the reaction were: a temperature of 37 °C, pressure at 3 bar, and 100 Mmol NADH in phosphate buffer solution with a pH of 7.0. It can also be seen that the free enzyme system provided a lower yield of CH_3_OH than the immobilization system. Therefore, enzyme immobilization could play a major role in the multi-enzymatic reactor system, not only by enhancing the stability and reusability of the enzymes but also by increasing the biocatalytic productivity of CO_2_ reduction. An even higher biocatalytic productivity of CO_2_ reduction was attained by Ren et al. [77], a metal–organic framework (MOF) incorporating ZIF-8 being utilized as the immobilization matrix to encapsulate FDH, GluDH, and CA enzymes. Compared with the pure polymeric and ceramic membranes, the MOF exhibited a higher CO_2_ adsorption capacity and at the same time could also effectively retain the biological activity of the enzymes and prevent them from denaturing [77,78]. Hence, the application of inorganic–organic hybrid microcapsules in immobilization support could help increase the performance of the EMR system for multi-enzymatic cascade catalysis of CO_2_.

It has also been reported by Gu et al. [72] that by combining the EMR system with a photocatalytic reactor, a quite high enzymatic CO_2_ conversion yield could be obtained, an integrated hollow fiber membrane reactor/photocatalytic reactor providing a CO_2_ reduction yield of approximately 80%. The aims of the system were to support enzyme immobilization together with the photochemical regeneration of NADH. It was noted that by utilizing an electron mediator (Rh complex) as a co-catalyst the photoregenerated NADH had a similar bioactivity with the fresh NADH, making it reliable for further use in the biocatalytic reduction of CO_2_. However, for a regular photocatalytic system, it is difficult to achieve a high yield of product from the multi-enzymatic cascade reduction of CO_2_. Aresta et al. [74] modified the structure of the TiO_2_ photocatalyst with a photosentisizer so that more light could be absorbed by the photocatalyst to obtain a high NADH regeneration yield. Ji et al. [21] developed a Z-scheme photocatalytic system to improve electron transfer efficiency and promote redox potentials, but the biocatalytic productivity of the photocatalytic system was still unable to achieve levels above 90%. Furthermore, the photochemical regeneration of NADH without an electron mediator could also lead to the synthesis of inactive NADH compounds, such as 1,6-NADH, instead of active 1,4-NADH. Therefore, the introduction of an electron mediator to the system is crucial for operating the multi-enyzmatic conversion of CO_2_ via a photocatalytic system to increase the selectivity of 1,4-NADH production. On the other hand, enzyme-coupled cofactor regeneration, which is usually applied by the EMR system, is more selective compared to the electrochemical and photochemical regeneration of NADH due to the involvement of a substrate with its enzymes, such as glutamate–glutamate dehydrogenase (GluDH), glucose–glucose dehydrogenase (GDH), phosphite–phosphite dehydrogenase (PTDH), and lactate–lactate dehydrogenase (LDH).

It has been reported that the electrochemical cell system has only been widely applied at laboratory scale and still has not yet been implemented in a large-scale production process [19]. Comparing the CO_2_ reduction yield obtained by the EMR and the photocatalytic reactor system with the electrochemical cell system, the latter has the lower biocatalytic productivity in multi-enzymatic cascade CO_2_ reduction. Biocatalytic CO_2_ reduction in the electrochemical cell would usually be initiated by the electrochemical regeneration of NADH. The NADH regenerated by the electrode would then be used to catalyze CO_2_ in the presence of oxidoreductases. One of the drawbacks of the electrochemical cell system is that the electrodes may also lose activity when they have been used in multiple cycles of operations [10], which could be a problem when it comes to catalyzing the electrochemical regeneration of NADH. This is because a constant amount of applied potential would have to be controlled and maintained to facilitate the regeneration of cofactors. The type of electrode used could also affect the yield obtained. For instance, Song et al. reported that the biocatalytic CO_2_ reduction yield obtained using CuNPs electrodeposited on a CF electrode is higher compared to a system with a pure CF electrode as the working electrode, the values for which were 22.8% and 10%, respectively [60,66].

## 5. Factors Affecting the Biocatalytic Productivity of Multi-Enzymatic Cascade Systems

### 5.1. Optimum Reaction Conditions

There are several process parameters involved in optimizing CH_3_OH production via the multi-enzymatic conversion of CO_2_, including pH, temperature, pressure, enzyme concentration, and NADH concentration. As for electrochemical cell and photocatalytic systems, the types of electrodes or semiconductors used may greatly affect the multi-enzymatic CO_2_ reduction. Due to the involvement of three dehydrogenases in one reactor unit for catalyzing the reduction of CO_2_ to CH_3_OH, the pH of the reaction solution (buffer) can easily affect the multi-enzymatic cascade reaction. Interestingly, each of the enzymes have their own optimum pH (i.e., the pH values at which they are most active). It is reported that the first reduction step, the reduction of CO_2_ to CHOOH catalyzed by FDH, is the most active at pH 6.0 [16,17], while the second and third reductions via FaldDH and ADH are favoured at pH 7.0 and 8.1, respectively [83]. Therefore, it is quite difficult to optimize the operating pH for the multi-enzymatic system. There is one alternative reported by Luo et al. [17] that could be applied, which is to carry out each of the reduction step separately at their respective optimum reaction conditions in order to achieve a high rate of CH_3_OH production. Jiang et al. [76] utilized three different pH values of buffer solution for catalyzing the enzymatic conversion of CO_2_—7.0, 7.5, and 8.0—and the yields obtained for each pH were 92.1%, 66.9% and 49.5%, respectively. Meanwhile, Sun et al. [81] compared the effect of pH on the activity of immobilized and free enzyme systems. Both systems had provided the highest yields of CH_3_OH at pH 7.0, but the immobilized enzyme system obtained a higher yield of CH_3_OH than the free enzyme system, approximately 50% and 10%, respectively. The optimum pH for the oxidation of NADH is also important because the multi-enzymatic reduction of CO_2_ requires the aid of the terminal electron to produce CH_3_OH. It was reported that NADH was not stable at a pH value lower than 4.0 [17]. Ren et al. [77] also studied the effect of pH on co-immobilized FDH, CA, and GluDH on ZIF-8 performance, and the optimum pH value of the reaction reported was 7.0, which yielded approximately 460% of CH_3_OH. For the bio-electrocatalytic system, Chen et al. [61] reported that an FDH-catalyzed CO_2_ reduction had an optimal activity at pH 7.0 and that activity decreased when the pH was lower than 6.0. Several studies have also reported that yields of CH_3_OH decreased when the pH value of the buffer solution was below 6.5 and above 7.5 [10,83,84]. This proves that even slight changes of pH could cause enzymes to lose their conformational shape and thereby reduce their biocatalytic ability. Nevertheless, this limitation can be overcome with enzyme immobilization.

The effect of temperature on multi-enzymatic cascade catalysis performance has also been evaluated. It has been reported that the optimum temperatures for FDH, FaldDH, and ADH were 37 °C, 37 °C, and 25 °C, respectively. Jiang et al. [76] proved that multi-enzymatic cascade catalysis proceeds better at 37 °C than at 25 °C due to the higher yield of CH_3_OH obtained, the values for which were about 92.1% and 30%, respectively. Sun et al. [81] reported that the immobilized and free enzyme systems were most active in a temperature range of 27–37 °C. However, the enzyme immobilization system provided a higher yield of CH_3_OH production compared to the free enzyme system at the operating temperatures applied. This is because the utilization of titania particles as the immobilization support gave protection to the enzymes from the slightly higher operating temperatures applied and increased the stability of the enzymes [81]. By immobilizing the enzymes in a ZIF-8 MOF, it was found that the activity of the multi-enzymatic system could be maintained at an even wider temperature range of 30–60 °C due to the higher mechanical strength and greater ability to retain the stability of the enzymes [77].

According to the reaction pathway of CO_2_ reduction by dehydrogenases, 3 mol of NADH would be needed to produce 1 mol of CH_3_OH. Therefore, the biocatalytic productivity of CO_2_ conversion could be dependent on the amount of NADH present in the multi-enzymatic reactor system. Song et al. [66] reported that the yield of CHOOH production increases as the concentration of NADH utilized increases. The amount of CHOOH obtained was higher when 3 mM NADH was used compared to the amount of CHOOH obtained when 1 mM NADH was used, the values for which were 5 mM and 3 mM, respectively. However, an excessive amount of NADH in the biocatalytic reactor could result in a low yield of CH_3_OH. Jiang et al. [76] reported that the yields of CH_3_OH obtained for a system utilizing 50, 100, and 150 Mmol NADH were 69%, 50%, and 32%, respectively, which showed that biocatalytic productivity decreases when higher concentrations of NADH are utilized. This is because large amounts of NAD^+^ would be generated from the multiple oxidations of NADH in producing high yields of CH_3_OH, which therefore could increase the tendency of CO_2_ conversion to go in the reverse (CH_3_OH → CO_2_) instead of the forward direction [12]. Li et al. [78] also tested the effects of NADH concentration on CHOOH production by varying NADH concentrations in the range of 0.5–2.8 mM, and the highest yield of CHOOH obtained was at 354% when 0.5 mM NADH was used, while CH_3_OH production dropped when 1–2.8 mM NADH was used. For enzymatic CO_2_ transformation in the electrochemical cell system, Barin et al. [64] reported that the optimum value of NADH concentration was about 0.45 mM to obtain a high yield of CHOOH at 300 min. For a photocatalytic system, it has been observed by Gu et al. [72] that the highest CHOOH production was achieved with 2 mM NADH, which was the optimal NADH concentration for the reaction. Hence, high biocatalytic productivity in CO_2_ conversion can be obtained by utilizing a low amount of NADH and a high concentration of NADH in the reactor can encourage the multi-enzymatic system to catalyze the reverse conversion of CH_3_OH to CO_2_.

### 5.2. Immobilization of Enzymes and Cofactors

For catalyzing the multi-enzymatic conversion of CO_2_, the orderly co-immobilization of the oxidoreductase system gives a better performance compared with the disordered enzymes in the membrane. Zhu et al. [42] reported that the amount of CH_3_OH synthesized by the ordered enzymes and co-enzymes with a ZIF-8@PVDF membrane is higher than the amount of CH_3_OH produced with a disordered enzymes system, the amounts being approximately 13.5 µmol and 6.6 µmol, respectively. However, the immobilized enzymes and cofactors could also provide lower biocatalytic productivity compared with the free enzymes because higher mass transfer resistances are expected for the immobilization system and the enzymes might also undergo conformational changes after they have been immobilized [79]. In addition, the immobilization of the enzymes could help prevent them from unfolding and denaturing due to pH, heat, organic solvents, and other operating parameters. The chief advantage of immobilization is that it allows for the reusability of enzymes. Li et al. [78] co-immobilized the oxidoreductases CA and GluDH in a modified metal–organic framework and reported that the yield of CHOOH production achieved was still high (86%) even after 10 cycles of reusing. CA aids CO_2_ hydration, while GluDH aids the regeneration of NADH.

### 5.3. Cofactor Regeneration

As the terminal electron and hydrogen donor for the multi-enzymatic reduction of CO_2_, the regeneration of NADH is essential to support continuous CH_3_OH production and reduce the supplement of NADH to the reactor. Another problem that may be faced in multi-enzymatic cascade catalysis without NADH regeneration is the NAD^+^ overly generated in situ, which could inhibit the reverse CO_2_ reduction by the dehydrogenases [83]. Therefore, it is very important to find an approach that could regenerate NADH effectively in the reactor system so that it could lead the system to achieve high CH_3_OH production from CO_2_. After reviewing each multi-enzymatic reactor system, it is clear that the cofactors can be regenerated in several ways for each multi-enzymatic reactor system. For the EMR system, one of the methods most used to regenerate the equimolar electron donor is by introducing a co-enzyme and co-substrate into the system. According to a majority of the reports on EMR systems, many of the systems applied the co-immobilization of glutamate dehydrogenase (GluDH) to regenerate NADH from its oxidized form, NAD^+^, and the yield of CH_3_OH obtained was higher for the systems that applied enzyme-coupled cofactor regeneration than the multi-enzymatic systems without any cofactor regeneration. The hypothesis has been proved by Ji et al. [75], in whose study the system with GluDH achieved a yield of CH_3_OH of approximately 33%, while CH_3_OH production recorded for the free enzyme system without the presence of GluDH was 28%. It was also highly recommended that the GluDH be co-encapsulated with the oxidoreductases through layer-by-layer assembly so that its activity and stability could be retained for several cycles of the operation along with the FDH, FaldDH, and ADH enzymes. The products generated from the co-substrate by the co-enzyme introduced could also be separated easily through co-immobilization in the EMR and enable easy recycling. There are other enzymes, also, that could be utilized for NADH regeneration, such as glucose dehydrogenase (GDH), xylose dehydrogenase (XDH), lactate dehydrogenase (LDH), among others. Marpani et al. [85] reported that the reduction rate (V_max_) of NAD^+^ to NADH via GDH is faster than the rate of NADH oxidation to NAD^+^ by ADH, the values for which are 6.3 µmol/mg.min and 4.7 µmol/mg.min, respectively. This is a good sign, as the NADH would be readily available to catalyze the CHOH to CH_3_OH during the third step of the multi-enzymatic cascade CO_2_ conversion. Another interesting finding has been made by utilizing phosphite dehydrogenase (PTDH) and phosphite in cofactor regeneration. This is that phosphate is produced from the oxidation of phosphite by PTDH, which therefore could be used as the buffer solution for the enzymatic reaction. PTDH could also give quite a high NADH regeneration yield, approximately 80% [80].

The regeneration of NADH using the photochemical approach is also attractive because it utilizes light irradiation to regenerate the equimolar electron donor, which makes it a sustainable and energy-saving process. An electron donor and a photocatalyst are usually required to conduct the photoregeneration of NADH. According to the existing photocatalytic method applied for regenerating NADH, TiO_2_ was the most frequently used and studied photocatalyst due to its having a higher chemical stability, a lower cost, and being less toxic compared to most of the metal oxide photocatalysts. It is also readily available [86]. A few types of electron donors have also been tested for the regeneration of NADH, including EDTA, TEOA, and H_2_O. It is reported that EDTA has a stronger complexing ability with TiO_2_ photocatalysts than TEOA and H_2_O, which makes it able to regenerate greater amounts of NADH to assist the biocatalytic reduction of CO_2_ to CH_3_OH. According to the report, the NADH generation yields obtained after 1.5 h utilizing EDTA, TEOA, and H_2_O as electron donors were 85%, 44%, and <35%, respectively [87]. Ji et al. [21] also studied the efficiency of electron donors in the photogeneration of NADH, and it was reported that TEOA provided a higher efficiency of NADH regeneration than H_2_O, approximately about 90%, while the NADH generation achieved by H_2_O was 50%. However, the challenge of the photoreduction of NAD^+^ to NADH is to improve the light adsorption ability of the photocatalysts, as this could lead to a low productivity of NADH regeneration. The modification of photocatalyst structure, band gap improvement, and the introduction of co-catalysts are necessary to achieve high yields and selectivity in the photochemical reduction of NAD^+^ to NADH [10].

NADH has also been regenerated successfully in electrochemical cell systems. Electrochemical NADH regeneration is achieved with electrodes that could supply the required reduction potential of NAD+ to NADH, which is about −1.1 V. The type of electrodes used could highly affect the yield of regenerated NADH. Barin et al. [62], utilizing Cu foam as the working electrode, obtained a yield of active NADH of approximately 80%. On the other hand, Song et al. [66] reported that a CF electrode modified with CuNPs was gave a higher 1,4-NADH production efficiency than a Cu foam electrode, which was about 92% without requiring any addition of an electron mediator. However, the activity of the electrodes may decrease after they have been used several times by the system, which could lead to the fluctuation of potentials and low yields of reduced NAD^+^ at the end. NAD^+^ concentration could also affect the yield of active NADH produced. Barin et al. [62] had reported that the yield of NADH increased as the concentration of NAD^+^ added increased. The highest active NADH production recorded after 4 h was 77.7 ± 2.0% when 1.1 mM NAD^+^ and −1.1 V applied potentials were supplied to the system, though it was reported that the adsorption and adhesion of nanofibers deposited onto the electrode surface (used for immobilizing FDH, FaldDH, and ADH) could obstruct the electron transfer pathway on the electrode and lower NADH regeneration. Due to the many challenges faced by the electrochemical and photochemical approach for regenerating NADH, applying the coupled enzymatic system is the most efficient way to regenerate NADH at present due to the higher selectivity and simplicity of the route compared to the electrochemical and photochemical cofactor regeneration methods. Thus, it is proved that with the involvement of cofactor regeneration, the EMR could obtain higher yields of CH_3_OH through CO_2_ multi-enzymatic catalysis.

## 6. Conclusions

The multi-enzymatic conversion of CO_2_ to CH_3_OH can be conducted successfully in the EMR, electrochemical cell, and photocatalytic reactor systems. However, the highest biocatalytic productivity via multi-enzymatic CO_2_ reduction was obtained by the EMR system. The EMR system had the simplest reactor design compared to the electrochemical cell and photocatalytic reactor systems, the membrane unit acting as the core element for the reactor system. In addition, enzyme immobilization and cofactor regeneration have a positive impact on multi-enzymatic cascade catalysis, making them great strategies for increasing the biocatalytic productivity of FDH-catalyzed CO_2_ conversion. With the involvement of CA and cofactor regeneration with the sequentially co-immobilized dehydrogenases in EMR, high yields of CHOOH and CH_3_OH have been obtained. It cannot be denied that the addition of CA improves the hydration rate of CO_2_. However, it might make the multi-enzymatic system even harder to control and optimized since each enzyme performs best in different reaction conditions. Hence, obtaining a general optimum reaction condition would be a challenge for the enzymatic cascade transformation of CO_2_. Apart from introducing a second enzyme and a second substrate for regenerating NADH, the electrochemical and photocatalytic approach could also be applied to regenerate the cofactors, but it has been reported that these two methods have the potential to regenerate inactive NADH and an electron mediator is required to increase the selectivity of active NADH regeneration. Moreover, several improvements would still have to be made to the electrochemical cell and photocatalytic reactor system to obtain high biocatalytic productivity. Thus, it can be concluded that the EMR system is the most suitable, facile, and flexible reactor system to catalyze the multi-enzymatic cascade conversion of CO_2_ at present, though the biocatalytic productivity of CO_2_ conversion could be further enhanced by developing mixed-matrix membranes that could provide robust immobilization support and at the same time facilitate the simultaneous adsorption and hydration of CO_2_.

## Figures and Tables

**Table 2:** Enzymatic reactor systems applied by researchers for catalyzing the reduction of CO_2_ and their biocatalytic performance.

Enzymatic Reactor Setup	Optimum Reactor Conditions	Immobilization Approach	Immobilization Matrix	Initial NADH Amount (mM)	${\mathrm{Yield}}_{{\mathrm{CH}}_{3}\mathrm{OH}}{/\mathrm{Yield}}_{\mathrm{CHOOH}}\;(\%)$	Faradaic Efficiency	NADH Regeneration	Ref.
Enzyme membrane reactor	PBS, pH 7.4, 30 °C, 1 h	Encapsulation	Co-immobilized in ZIF-8	n.a.	460.0	-	Co-immobilization of glutamate dehydrogenase (GluDH) and PEI	[77]
			Free enzyme system	n.a.	100.0	-		
Enzyme membrane reactor	18 mL, PBS, pH 7, 27–37 °C, 24 h	Physical adsorption	Co-immobilized in polystyrene particles	0.05	50.0	-	Co-immobilization of GDH	[79]
Enzymatic membrane reactor	0.6 mL, PBS, pH 6.5, 37 °C, 3 h, 5 bar	Encapsulation	Phospholipid–silica nanocapsules (NPS)	100	45.2	-	Co-immobilizing phosphite dehydrogenase (PTDH)	[80]
Enzyme membrane reactor	2 mL, Tris-HCl, pH 7, 27–37 °C, 4 h	Encapsulation	Co-immobilization in protamine-templated titania	25	60.0	-	-	[81]
Enzyme membrane reactor	2 mL, PBS, pH 7	Encapsulation	Silica sol–gel	25	91.2	-	-	[12]
Polyelectrolyte-doped hollow nanofibers membrane reactor	2 mL, PBS, pH 7, 20 °C, 10 h	Encapsulation	Poly(allylamine hydrochloride) (PAH)-doped PU nanofibers	0.2	103.2	-	Co-immobilization of GluDH	[75]
			Free enzyme system	0.2	36.2	-		
Flat-sheet polymeric membrane reactor	4 mL, Tris-HCl, pH 7, 20 °C, 30 min, 2 bar		Free enzyme system	50	3.2	-	Co-immobilization of GluDH	[17]
		Fouling induced enzyme immobilization (involving entrapment and adsorption)	Co-immobilization system	50	3.0	-		
			Sequential immobilization system	50	4.2	-		
Ultrathin hybrid enzyme membrane reactor	1 mL, PBS, pH 7, 37 °C, 3 bar	Entrapment	Gelatin modified with catechol groups (GelC)–silica hybrid microcapsules	50	71.6	-	-	[82]
			Free enzyme system	50	35.5	-	-	
Photo-enzymatic reactor	20 mL, EDTA–NaOH buffer solution, pH 7, 37 °C, 4.5 h	Encapsulation	Polyethylene hollow fiber membrane (PE HFM)	2	81.7	-	Regenerated photochemically by utilizing TiO_2_ photocatalyst, EDTA as electron donor and [Cp*Rh(bpy)(H_2_O)]^2+^ as co-catalysis	[72]
Photocatalyic reactor	10 mL, PBS, pH 7.0, 5 h	Physical adsorption	Antimonene (AM)–electron mediator (M, Cp*Rh(phen)Cl)–black phosphorus (BP) hybrid nanosheet (AM/M/BP HNS)	-	89.0	-	Regenerated photochemically by utilizing Z-scheme electron transfer in AM/M/BP HNS and TEOA as electron donor	[21]
Photocatalytic reactor	10 mL, TEOS, pH 7.0, 1 h	Encapsulation	Ca alginate beads	-	n.a.	n.d.	Regenerated photochemically by utilizing [CrF_5_(H_2_O)]^2−^@TiO_2_ photocatalyst, [Cp*Rh(bpy)H_2_O]Cl_2_ as electron mediator and water (H_2_O) as electron donor	[74]
Electrochemical reactor	25 mL per compartment cell, applied potential of −1.2 V, carbon felt as working electrode, PBS, pH 7.6, 4 h	Physcial adsorption	Alginate–silicate hybrid gel	-	n.d	10.0	-	[60]
Electrochemical H-shaped cell	20 mL per half-cell, Cu foam electrode, Nafion 117 membrane, PBS, pH 7.0, 25 °C, 5 h	Physcial adsorption	Modified electrospun polystyrene fibers	-	n.d.	n.d.	Regenerated electrochemically by utilizing Cu foam electrode, 0.95 mM NAD^+^ and applying constant potential at −1.1 V	[62]
Electrochemical reactor	5 mL, CuNPs/CF electrode, 0.1 M PBS, pH 6.0, 5 h	Physical adsorption	Cu nanoparticles (CuNPs)	3	n.d.	22.8	Regenerated electrochemically by utilizing CuNPs electrodeposited on CF electrode, 1.1 mM NAD^+^ and applied potential at −1.2 V	[66]
Electrochemical cell	10 mL, Rh-FTO electrode, Tris buffer, pH 7.0, 1 h	Encapsulation	NU-1006	-	79.0	n.d.	Regenerated electrochemically by utilizing Rh-FTO electrode, 1 mM NAD^+^ and applied potential at −1.1 V	[61]
Enzyme membrane reactor	4 mL, Tris-HCl, pH 7, 30 min	Fouling-induced immobilization	Polypropylene modified cellulose membrane	5	24.5	-	Co-immobilization of glucose dehydrogenase (GDH)	[83]
	4 mL, mixture of choline and L-glutamic acid ([CH][Glu]) ionic liquid solution, pH 7, 30 min			5	85.8	-		
Enzyme membrane reactor	250 mL, PBS, pH 7, 37 °C, 3 bar	Encapsulation	Silica sol–gel	100	92.1	-	-	[76]
Enzyme membrane reactor	6 mL, PBS, 25 °C, 5 bar, 6 h	Encapsulation	HKUST-1@PEI(100)-MIL-101(Cr)	0.1	353.9	-	Co-immobilization of GluDH	[78]
Enzyme membrane reactor	10 mL, PBS, 6 h	Fouling-induced immobilization	Ordered co-immobilization of enzymes and co-enzymes in ZIF-8@PVDF	10	40.5	-	Co-immobilization of GluDH	[42]
			Disordered immobilization of enzymes in ZIF-8@PVDF	10	19.8	-		
			Free enzymes and co-enzymes in solution	10	18.0	-		

## Data Availability

Not applicable.
